# Adipose-derived mesenchymal stem cells attenuate acute lung injury and improve the gut microbiota in septic rats

**DOI:** 10.1186/s13287-020-01902-5

**Published:** 2020-09-07

**Authors:** Junyi Sun, Xianfei Ding, Shaohua Liu, Xiaoguang Duan, Huoyan Liang, Tongwen Sun

**Affiliations:** 1 General Intensive Care Unit, The First Affiliated Hospital of Zhengzhou University, Zhengzhou Key Laboratory of Sepsis, Henan Key Laboratory of Critical Care Medicine, Henan Engineering Research Center of Critical Care Medicine, Zhengzhou, 450052 China; 2 Academy of Medical Sciences of Zhengzhou University Translational Medicine Platform, Zhengzhou, 450052 China

**Keywords:** Acute lung injury, Mesenchymal stem cells, Sepsis, Cecal ligation and puncture, Gut microbiota

## Abstract

**Background:**

We hypothesized that adipose-derived mesenchymal stem cells (ADMSCs) may ameliorate sepsis-induced acute lung injury (ALI) and change microorganism populations in the gut microbiota, such as that of Firmicutes and Bacteroidetes.

**Methods:**

A total of 60 male adult Sprague-Dawley (SD) rats were separated into three groups: the sham control (SC) group, the sepsis induced by cecal ligation and puncture (CLP) group, and the ADMSC treatment (CLP-ADMSCs) group, in which rats underwent the CLP procedure and then received 1 × 10^6^ ADMSCs. Rats were sacrificed 24 h after the SC or CLP procedures. To study the role of ADMSCs during ALI caused by sepsis and examine the impact of ADMSCs on the gut microbiome composition, rat lungs were histologically evaluated using hematoxylin and eosin (H&E) staining, serum levels of pro-inflammatory factors were detected using enzyme-linked immunosorbent assay (ELISA), and fecal samples were collected and analyzed using 16S rDNA sequencing.

**Results:**

The serum levels of inflammatory cytokines, tumor necrosis factor (TNF)-α and interleukin (IL)-6, were significantly increased in rats after the CLP procedure, but were significantly decreased in rats treated with ADMSCs. Histological evaluation of the rat lungs yielded results consistent with the changes in IL-6 levels among all groups. Treatment with ADMSCs significantly increased the diversity of the gut microbiota in rats with sepsis. The principal coordinates analysis (PCoA) results showed that there was a significant difference between the gut microbiota of the CLP-ADMSCs group and that of the CLP group. In rats with sepsis, the proportion of *Escherichia*–*Shigella* (*P* = 0.01) related to lipopolysaccharide production increased, and the proportion of *Akkermansia* (*P* = 0.02) related to the regulation of intestinal mucosal thickness and the maintenance of intestinal barrier function decreased. These changes in the gut microbiota break the energy balance, aggravate inflammatory reactions, reduce intestinal barrier functions, and promote the translocation of intestinal bacteria. Intervention with ADMSCs increased the proportion of beneficial bacteria, reduced the proportion of harmful bacteria, and normalized the gut microbiota.

**Conclusions:**

Therapeutically administered ADMSCs ameliorate CLP-induced ALI and improves gut microbiota, which provides a potential therapeutic mechanism for ADMSCs in the treatment of sepsis.

## Introduction

Sepsis is a public health problem characterized by life-threatening organ dysfunction due to maladjustment of the host response to infection [1, 2]. In the intensive care unit (ICU), the global mortality rate of sepsis is close to 25% [3, 4]. Sepsis involves the damage of various important organ functions, including brain, kidney, and heart functions [5–8]. ALI can easily occur in the early stage of sepsis due to the involvement of the lungs in suppurative inflammation [9]. And there are many potential pathogens that can cause sepsis [10]. More importantly, there is no drug or treatment that can effectively cure sepsis [11]. Therefore, it is necessary for us to develop new therapeutic methods for ALI treatment.

In recent years, ADMSCs have been used as a new treatment strategy for diseases related to inflammation and tissue damage [12–15]. MSCs are easy to obtain and possess immunomodulatory therapeutic properties and genetic stability [16]. MSCs can actively balance cytokines [17], stimulate the phagocytosis of monocytes and macrophages [18, 19], promote bacterial clearance, and prevent ALI and organ dysfunction [20]. Our previous studies also found that ADMSCs attenuate sepsis-related ALI by releasing sTNFR1 [21]. ADMSCs can alleviate inflammation and oxidative stress [22, 23], and many studies have confirmed the therapeutic effect of mesenchymal stem cells (MSCs) in sepsis animal models [24–27]. Furthermore, MSCs can inhibit the MAPK pathway activation during sepsis [28]. In recent years, studies have found that the mechanisms by which MSCs exert their therapeutic effects are manifold, but in general, MSCs are considered to be able to form a balanced inflammation and regeneration microenvironment in damaged tissues under severe inflammation [29], which provides an idea for our research. Recent studies have found that ADMSCs and cord-derived MSCs show therapeutic effects on inflammation and maintain the balance of the gut microbiota after early cell injection [30].

The gut microbiota is a complex microbial community that has been shown to play a major role in health and disease [31]. The gut microbiota consists of microorganisms that reside in the host gastrointestinal tract. These microorganisms provide protection against pathogen colonization and invasion through appropriate responses, leading to inflammation or tolerance. Humans have nearly 100 trillion gut bacteria that are vital for good health. Millions of years of coevolution have shaped the symbiotic relationship between humans and microorganisms, in which the gut bacteria play a crucial role in the metabolism of human nutrients, while the human gut provides a nutrient rich environment for the bacteria. Therefore, an imbalance in the gut microecology leads to the occurrence or aggravation of disease [32–37]. Dysfunction of the intestinal epithelium, dysfunction of the immune system, and translocation of enteropathogenic bacteria are considered to be the key factors leading to infectious complications and multiple organ dysfunction syndromes. The gut is considered to be a driving factor for sepsis and multiple organ dysfunction syndromes [38]. As most of the microorganisms in the human intestine are anaerobic, once excreted with feces, their biological activity is lost [39]. Therefore, the composition of the intestinal microflora detected by clinical fecal samples through bacterial culture does not fully reflect the overall picture of the human intestinal microecology. To address this problem, in animal experiments, feces collected directly from the intestine can be used for microbiological analysis to better reflect the overall picture of the intestinal microecology. Because of the barrier function of intestinal mucosa to bacteria in physiological state, it can avoid the interference of harmful bacteria to other tissues and organs of the human body [40]. But the occurrence of sepsis will destroy the intestinal barrier and cause the imbalance of intestinal flora and the translocation of intestinal endogenous bacteria [41, 42], which will lead to the lung becomes the most common and important damaged organ [43]. Therefore, intestinal endogenous bacterial infection is an important reason of ALI. In addition, studies on the relationship between pulmonary microbiome and disease are also increasing [44–49]. The studies found that healthy lungs also have microbiome [50], and the state of pulmonary microbiome changes with the severity of the disease [51, 52]. Some studies have found that the intestinal and pulmonary microbiome of severe patients have obvious changes after passing through the gut-lung axis, which has an impact on sepsis-associated ALI or acute respiratory distress syndrome (ARDS) [45, 53]. The composition of the microbiome in healthy lungs is determined by the migration, clearance balance, and growth rate of microorganisms [54, 55]. At present, the repertoire of the airway microbiome of human beings has been established [56], which is convenient for comparative analysis with other microbial components. Some studies have shown that early use of antibiotics is beneficial to patients with severe sepsis [57], but the high mortality caused by sepsis and acute organ injury remains to be solved. However, the use of drugs, such as antibiotics, may change the microbiome in the body and disrupt the relationship between microbiota and host [58]. And antibiotic treatment reduced the number of bacteria in the blood of sepsis mice, which will interfere the efficacy of MSCs [59]. Steroids can also affect the microbiome [51]. Some animal experiments and clinical studies have found that the bronchoalveolar lavage fluid (BAL) can be extracted or lung tissue can be sequenced to observe characteristics of the lung microbiome, which greatly reduces the impact on host microbiome [48, 52, 60]. In addition to sequencing methods, the microbiome can also be identified by conventional culture methods [52, 61]. 16S rDNA sequencing is a method for high-throughput and rapid identification of bacterial species [62]. In this study, we used 16S rDNA technology to observe the effect of early treatment with ADMSCs on the intestinal microecology of rats with sepsis, and we compared the general changes in intestinal bacteria between different groups.

In this study, SD rats with sepsis induced by CLP were used to test whether treatment with ADMSCs could improve the ALI caused by sepsis and change the intestinal microbiome.

## Materials and methods

### Experimental materials

#### Animals

A total of 60 male adult SD rats (200–250 g, 6–8 weeks old) were purchased from Charles River (Beijing, China, http://www.vitalriver.com) and placed in the temperature control and light control room (20–25 °C, 1:1 light dark cycle) for free feeding and drinking. We used the method of computer random assignment for grouping. And the animals used in the experiment are the same batch of rats. Rats were randomly divided into three groups (20 rats in each group): the SC group, the CLP group, and the CLP-ADMSCs treatment group. The SC and CLP groups were injected with normal saline, and the CLP-ADMSCs group was injected with 1 × 10^6^ ADMSCs through the tail vein 1 h after CLP operation. Rats were killed 24 h after the operation and their serum, feces, and lung tissue samples were collected for analysis. This study was conducted in accordance with the principles described in the National Institutes of Health (NIH) guidelines for the care and use of laboratory animals (http://grants1.nih.gov/grants/olaw/). All experiments were approved by the ethics review committee of life sciences at Zhengzhou University.

#### Main reagents

Ten percent chloral hydrate was purchased from SLB-bio (Beijing, China). ADMSCs were purchased from Cyagen (China). ELISA kits of rat IL-6 and TNF-α were purchased from R&D Systems (USA). 0.25% ethylenediaminetetraacetic acid (EDTA) trypsin was purchased from SLB-bio. Phosphate-buffered saline (PBS) and the E.Z.N.A.® Stool DNA Kit were purchased from HyClone Laboratories (USA) and Omega Bio-tek (USA), respectively. The Library Quantification Kit for Illumina® was purchased from Kapa Biosciences (Woburn, USA). Fetal bovine serum and penicillin-streptomycin solution (100×) were purchased from Gemini Bio (USA) and SLB-bio (Beijing, China), respectively. Dimethyl sulfoxide (DMSO) and isopropyl alcohol were purchased from Sigma (USA) and Tianjin Hengxing Chemical Reagent Manufacturing Co., Ltd. (Tianjin, China), respectively.

#### Main instruments

The ultra-clean workbench and carbon dioxide incubator used in this study were produced by Thermo Fisher Scientific (USA). The water bath was manufactured by Zhongkelianyi Technology (Beijing, China). The manufacturers of the optical microscopes and micro-samplers were Olympus (Japan) and Eppendorf (USA), respectively. The cell culture plates, cell cryopreservation tubes, and culture flasks were produced by Corning (USA). The 2100 Bioanalyzer system was manufactured by Agilent (Santa Clara, USA). The manufacturers of the − 20 °C and − 80 °C refrigerators were Haier (China) and Thermo Fisher Scientific, respectively.

## Methods

### CLP-induced sepsis rat model

The CLP model was simulated based on previous literature [42]. Briefly, after intra-peritoneal injection of 10% chloral hydrate (350 mg/kg), the rat abdomen was scraped and thoroughly cleaned with compound iodine, and surgery was performed on a sterile plate. The laparotomy was performed under anesthesia in rats, and then, a 2.0-cm incision was made at the midline of the abdomen. The cecum was ligated and punctured twice with a 10-mL syringe needle at the blind end, and a certain quantity of feces was squeezed out and placed in the peritoneal cavity. The abdominal muscle layer was sutured with sterile 5+0 surgical sutures, and the skin was sutured with sterile 3+0 surgical sutures. Antibiotics were not given to the rats during abdominal closure in order to obtain the simple therapeutic effect of MSCs. All rats were immediately placed in a warm environment after surgery and subcutaneously injected with pre-heated normal saline (50 mL/kg) for fluid resuscitation [63]. We choose subcutaneous injection of normal saline can be absorbed more quickly, accelerate the recovery of animals, and prevent dehydration caused by surgery. Rats in the SC group were treated similarly, but the cecum was not ligated or punctured.

### ADMSC culture and labeling

Second-generation ADMSCs extracted from rat groin fat were purchased from Cyagen Biotechnology (Yangzhou, China, http://www.cyagen.com/cn/zh-cn/). Cells were cultured according to the manufacturer’s instructions. Flow cytometry analysis showed that CD29, CD90, and CD44 were positively expressed, while CD45, CD11b, and CD34 were negatively expressed in the ADMSCs. Penicillin-streptomycin solution (100×) was used to prevent bacterial contamination in cell culture. The cells were cultured in medium (25 μmol/L) to the fourth passage and maintained for 24 h for later use.

### ELISA analysis

Blood samples were collected from the abdominal aorta of the rats. After 30 min, serum was collected and centrifuged (2500 rpm, 15 min). Serum samples were stored at − 80 °C for ELISA analysis.

### Histological analysis of rat lungs

Rat chests were opened to expose the heart, the inferior vena cava was cut off, and a perfusion needle was inserted into the apex of the heart. One hundred to 200 mL of cold saline was rapidly pushed into the left ventricle and aorta. When the lung turned white, half of the lung tissue was extracted and put into 4% paraformaldehyde for histological analysis, and the other half was rapidly frozen in liquid nitrogen. The lung tissue was embedded in paraffin and sliced using a slicer. H&E staining was used to evaluate the lung tissue from the rats. The procedure has been described in detail in the related literature [64]. The lung tissue was resected at a size of 5 μm and sectioned. H&E staining was used to analyze the lung tissue.

### Sequencing and analysis of the gut microbiota

Twenty-four hours after the operation, the rat feces from each group was squeezed into 2-mL cryopreservation tubes, labeled uniformly, and stored at − 80 °C. The 16S rDNA was sequenced using a NovaSeq PE250 platform (Illumina) after the bacterial DNA was extracted from the rat intestine (Hangzhou Lianchuan Biotechnology Co., Ltd., Hangzhou, China). In order to ensure the efficiency and quality of the DNA extracted from the different microorganism sample sources, the best DNA extraction method for the microorganism group was selected and the quality of the extracted DNA was detected using agarose gel electrophoresis. Meanwhile, DNA was quantified using an ultraviolet spectrophotometer. The electrophoresis detection time of the polymerase chain reaction (PCR) products was generally within 48 h, and the bands were irregular or even disappeared after 48 h. LC-bio uses AMPure XT beads (Beckman Coulter Genomics, Danvers, MA, USA) to purify PCR products and Qubit (Invitrogen, USA) for quantification. The size and quantity of the amplification library were evaluated using the library quantitative kit of the 2100 Bioanalyzer and Illumina (Kapa Biosciences, Woburn, MA, USA), respectively. Alpha diversity was used to analyze the complexity of the sample species diversity. The five indicators, including Chao1, Observed species, Goods coverage, Shannon, and Simpson, in the samples, were calculated using the QIIME2 microbiome bioinformatics platform (https://qiime2.org). Other diagrams were implemented using R-package (v3.5.2) [65–67]. 16S rDNA high-throughput sequencing technology was used to analyze the intestinal microecological diversity, as well as to identify and compare the differences in the composition of the gut microbiota from the different treatment groups.

### Statistical analysis

All results were expressed as the mean ± standard error. One-way analysis of variance (ANOVA) and Tukey’s post hoc test were used to evaluate the differences among the three groups. The difference was statistically significant when *P* < 0.05. We used SPSS Statistics 21.0 (IBM, Chicago, IL) software to analyze the data.

## Results

### ADMSCs can improve the 24-h survival rate of rats, reduce lung injury, and reduce TNF-α and IL-6 serum levels

The cecum of CLP-induced sepsis rats was congested and necrotic, while the cecum of rats from the CLP-ADMSCs group was significantly improved (Fig. S1, macroscopic view of the rat model). In addition, the 24-h mortality rates of the SC group, CLP group, and CLP-ADMSCs group were 0% (0/20), 40.0% (8/20), and 25.0% (5/20), respectively. The 24-h mortality rate of the SC group compared with that of the CLP group (*P* = 0.003). The 24-h mortality rate of the CLP group compared with that of the CLP-ADMSCs group (*P* = 0.501). The 24-h mortality rates and *P* values for each experimental group are shown in Table 1.

**Table 1:** 24-h mortality of animals in each group

Group	Fatality rate (deaths/total)	***P*** value
SC	0% (0/20)	0.003 ^a^
CLP	40.0% (8/20)	0.501^b^
CLP-ADMSCs	25.0% (5/20)	

^a^Compared with CLP group

^b^Compared with CLP-ADMSCs group

**Fig. 1:**
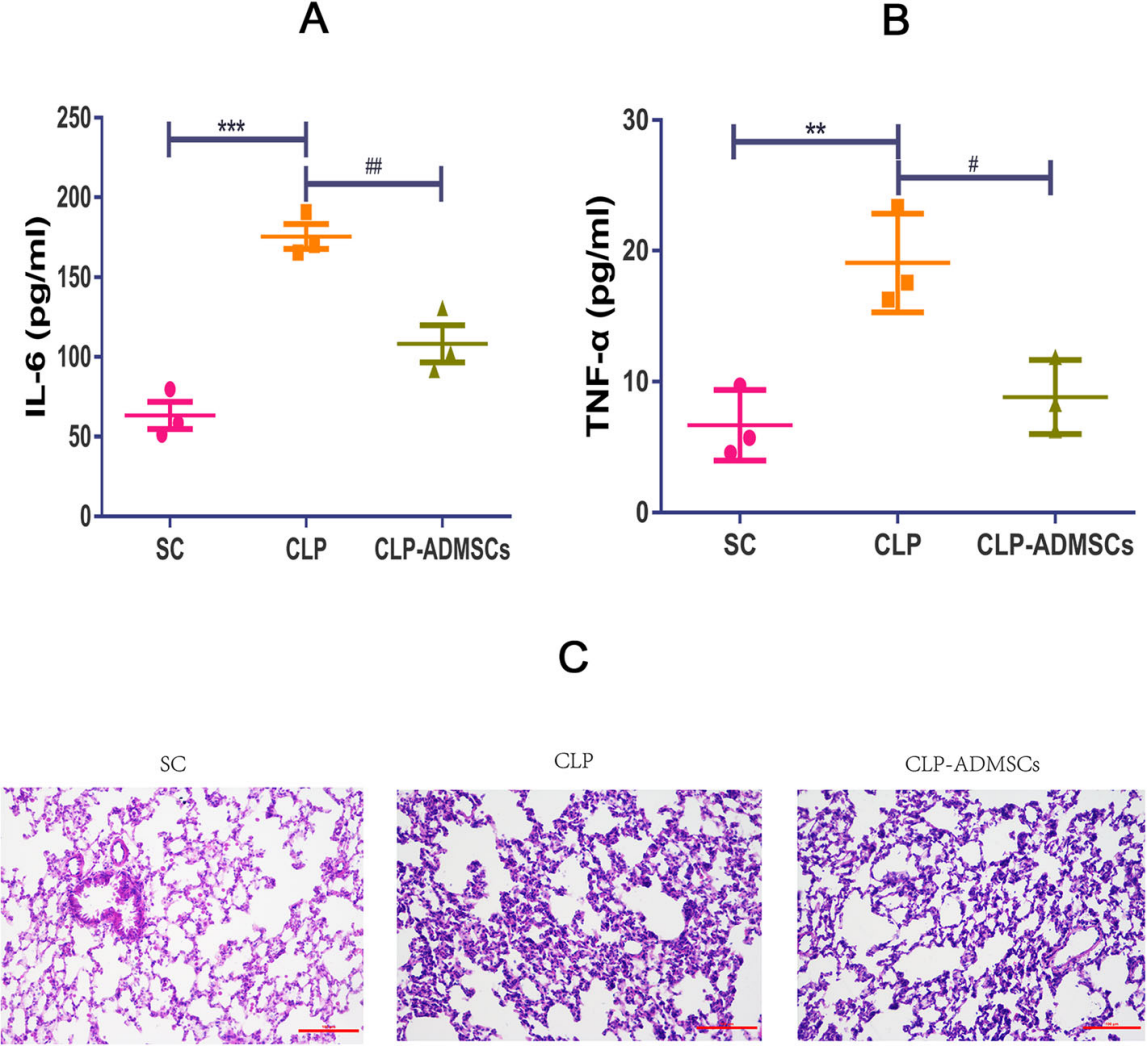
Effect of adipose-derived mesenchymal stem cells (ADMSCs) on systemic inflammatory response in rats with cecal ligation and puncture (CLP)-induced acute lung injury (ALI). The serum levels of IL-6 (**a**) and TNF-α (**b**) were measured using enzyme-linked immunosorbent assay (ELISA). *Compared with the sham control (SC) group; ^#^compared with the CLP group; *, ^#^*P* < 0.05; **, ^##^*P* < 0.01; ***, ^###^*P* < 0.001. **c** Effect of CLP and ADMSCs on 24-h lung histology. Hematoxylin and eosin staining showed that inflammatory infiltration, edema, and hemorrhage of rat lung tissue from the CLP group were significantly enhanced. Treatment with ADMSCs improved these anomalies (bar = 100 μm)

ELISA was used to detect the levels of IL-6 and TNF-α in 24-h serum. The results showed that the serum levels of IL-6 (*P* < 0.001) and TNF-α (*P* < 0.01) were significantly increased, and treatment with ADMSCs significantly reduced the levels of IL-6 (*P* < 0.01) and TNF-α (*P* < 0.05), as shown in Fig. 1a, b.

As shown in Fig. 1c, H&E staining revealed increased inflammatory infiltration, edema, and hemorrhage in the CLP group, and treatment with ADMSCs improved these abnormal pathological manifestations.

### Alpha diversity of the gut microbiota in rats

**Fig. 2:**
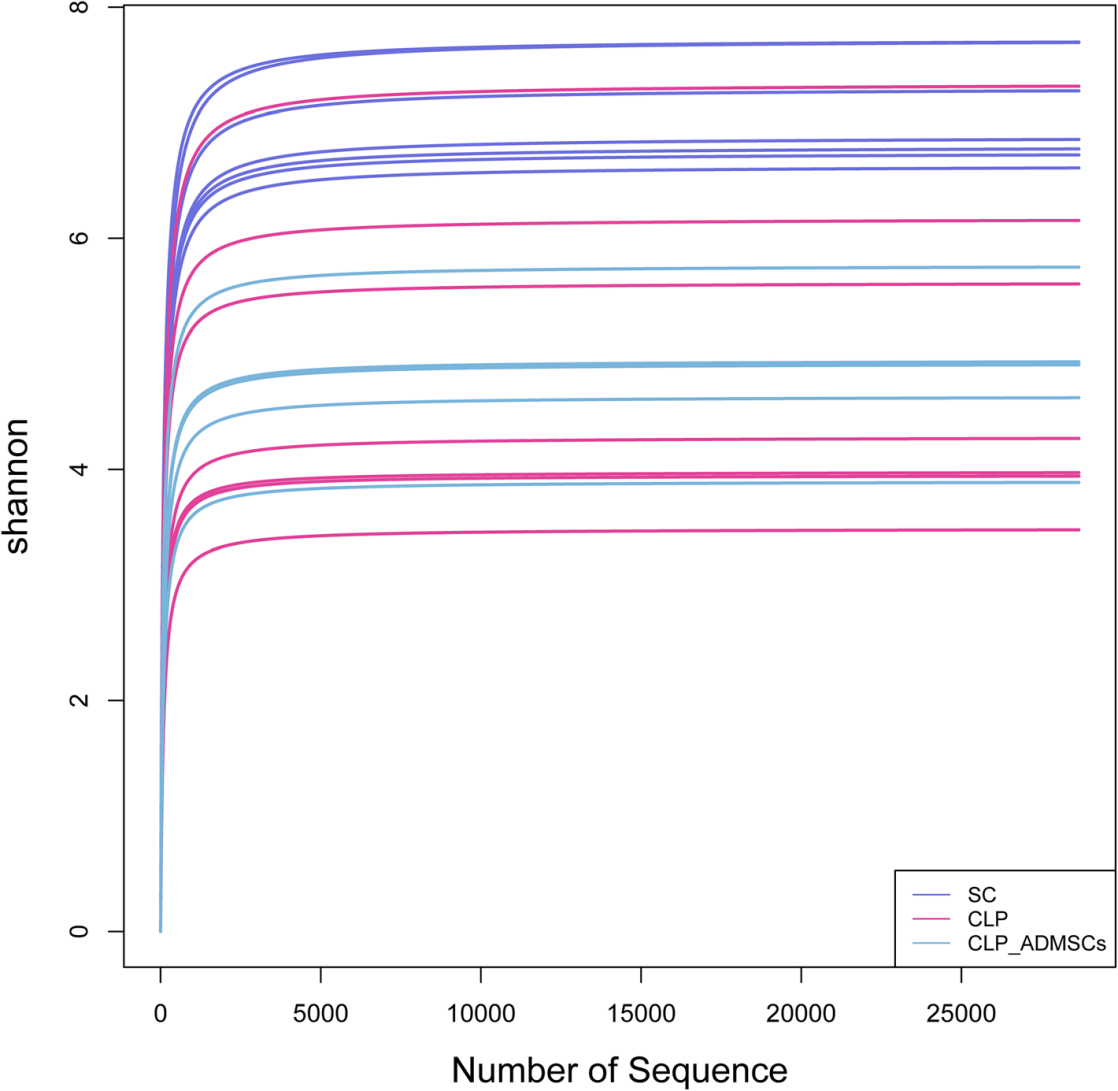
Abundance analysis of intestinal microecology. The abscissa represents the number of randomly selected sequences. The ordinate represents the number of operational taxonomic units (OTUs) observed

The abundance curve shown in Fig. 2 was used to evaluate whether the detected 16S rDNA sequences covered all microbial species in the sample. The abscissa in the graph represents the number of randomly selected sequences, and the ordinate represents the number of observed operational taxonomic units (OTUs). A short sample curve indicated a small number of sequences. A plateau in the curve indicated that the sequencing was saturated and increasing the amount of data would not obtain new OTUs; however, if the curve did not plateau, the sequencing was not saturated and increasing the amount of data would yield more OTUs. It can be seen from Fig. 2 that there is a turning point in the richness curve of each sample when the sequence number was about 3000 and that the Shannon curve tends to be flat. This proves that the amount of sequencing data from each group of samples was saturated.

### Beta diversity of the gut microbiota in rats

Beta diversity refers to the species diversity among different environmental communities. Together, beta diversity and alpha diversity constitute the overall diversity or the biological heterogeneity of a certain environmental community. To observe differences between samples, beta diversity analysis usually starts with the calculation of the distance matrix between environmental samples, which includes the distance between any two samples, mainly through principal component analysis (PCA), principal coordinates analysis (PCoA), clustering analysis (UPGMA), multidimensional scaling (NMDS), analysis of similarities (ANOSIM), permutational multivariate ANOVA (PERMANOVA, also known as Adonis), and other methods.

**Fig. 3:**
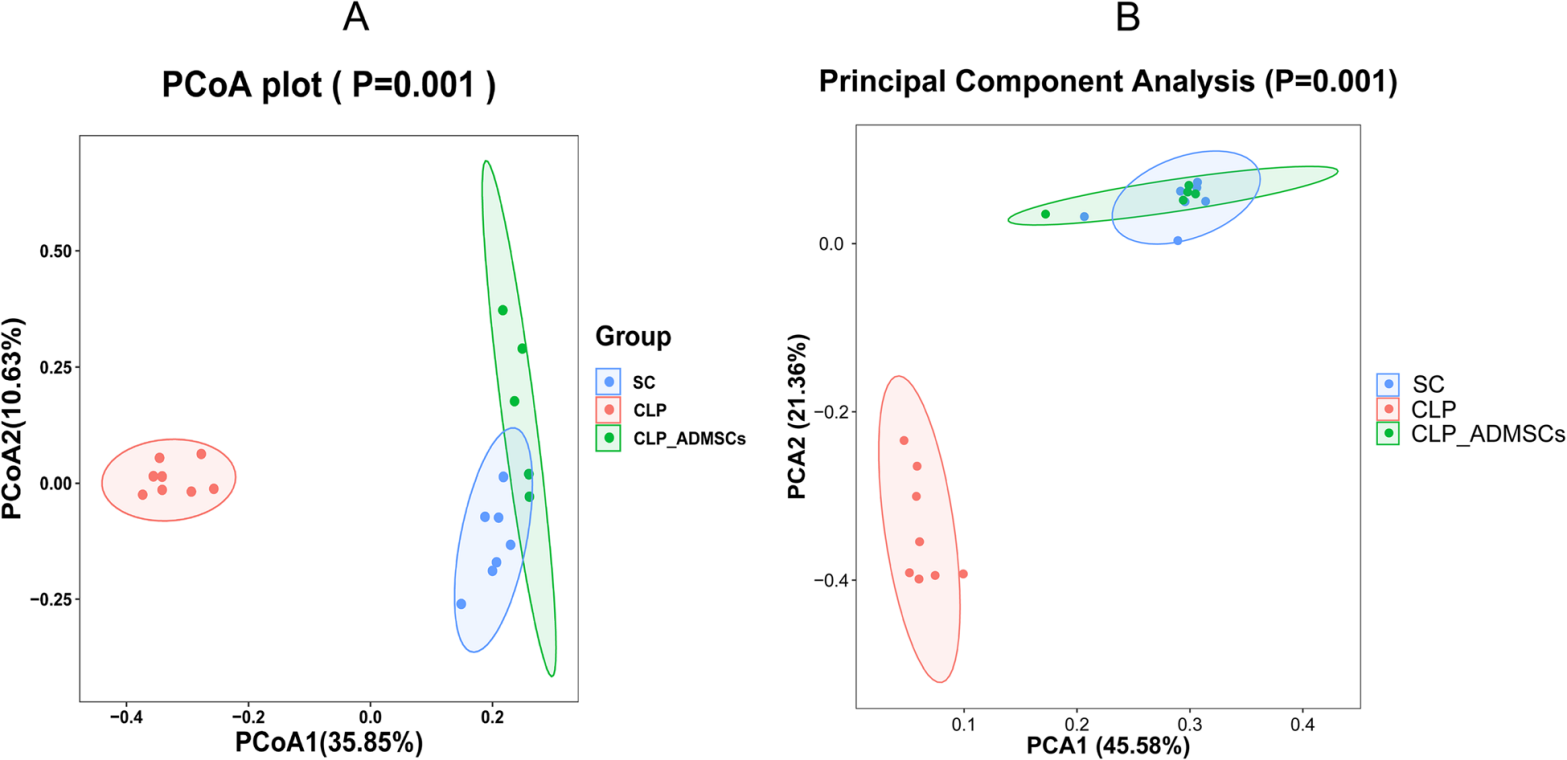
**a** Principal coordinates analysis (PCoA) based on unweighted UniFrac values of rats from the different treatment groups. **b** Principal component analysis (PCA) of rats from the different treatment groups

PCoA analysis is a visual method to study the similarities or differences between data. PCoA can also be used to observe the differences between individuals or groups. In the two-dimensional scatter diagram of the PCoA in Fig. 3a, the different groups are represented by different colors. The distance between the samples represents the similarity between the samples, where samples that are closer together are more similar in microbial composition. In the scatter plot, rat samples from the same group gathered together, indicating that the similarity between the gut microbiota of rats from the same group was very high. The greater the distance between different rat groups, the lower the similarity between the samples. In Fig. 3, the degree of contribution by PCoA1 and PCoA2 is 0.3585 and 0.1063, respectively, which can be used to distinguish the intestinal microflora of the rats from the different treatment groups. Therefore, based on the unweighted PCoA in Fig. 3a, the CLP group was significantly different from the control group and, after ADMSC treatment, the overall similarity between the SC group and the CLP-ADMSCs group was restored. In addition, the PCA in Fig. 3b was consistent with the PCoA results, indicating that ADMSCs had a positive impact on the number and diversity of intestinal microorganisms. The PCA showed that the structure of the gut microbiota changed significantly after CLP treatment compared to the SC group, but there was little difference between ADMSC-treated rats and the SC group.

### Structure analysis of the gut microbiota in rats

**Fig. 4:**
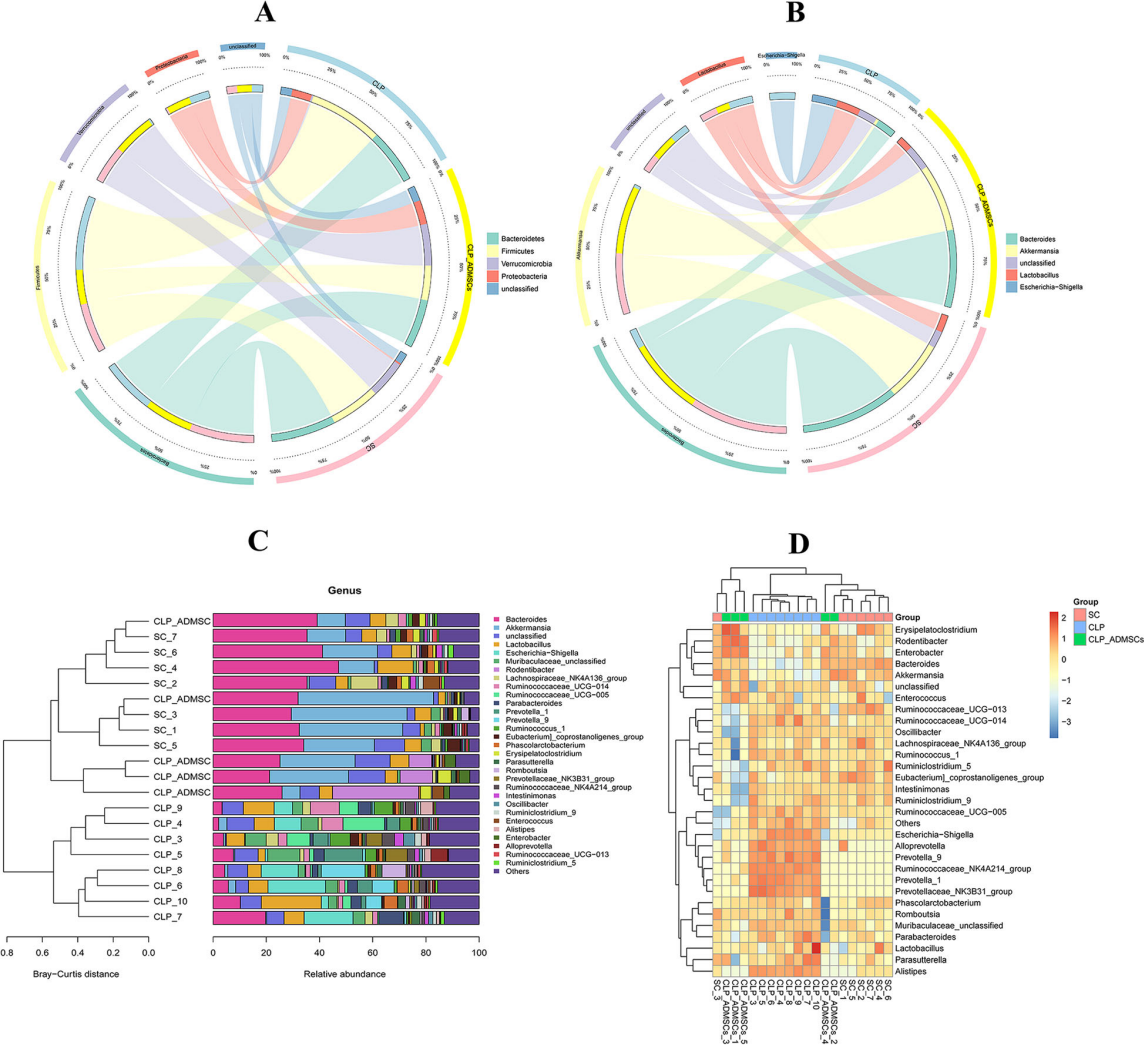
**a** Distribution of phylum in the gut microbiota of rats from the different treatment groups. **b** Distribution of genus in the gut microbiota of rats from the different treatment groups. **d** Cluster graphs. Left: The Bray-Curtis distance clustering tree structure. Closer sample clustering and shorter branches indicate a higher degree of similarity in the sample species composition. Right: The relative abundance distribution map of each sample at the gate level. A larger proportion indicates higher abundance. **d** Heat map. The gradient colors from blue to red reflect the change in abundance from low to high. Colors closer to blue indicate a lower abundance; colors closer to red indicate a higher abundance

In order to further explore the effect of treatment with ADMSCs on the composition of the rat gut microbiota, we statistically analyzed the phylum and genus of the microorganisms in the gut flora. Figure 4a shows the distribution of Bacteroidetes, Firmicutes, Verrucomicrobia, and Proteobacteria. Compared to the CLP group, the abundance of Proteobacteria decreased, and the proportion of Verrucomicrobia increased in the SC group, indicating that CLP had a great influence on the composition of the intestinal microflora. On the contrary, treatment with ADMSCs reduced the intestinal disturbance and the abundance of Proteobacteria. Figure 4b shows the genus distribution. Under normal conditions, the abundance of *Escherichia*-*Shigella* was very low, while that of the CLP group was significantly increased. The production of lipopolysaccharide (LPS) by *Escherichia*-*Shigella* destroys the balance of the gut microbiota [68–70]. Figure 4c shows that treatment with ADMSCs increased the abundance of *Akkermansia*, which can regulate the thickness of the intestinal mucosa, so as to maintain the intestinal barrier and provide a protective effect [71–73]. The results showed that treatment with ADMSCs could regulate and improve the microflora of rats with sepsis and had a tendency to restore its abundance. Figure 4d shows that the bacterial abundance of the CLP-ADMSCs group is close to that of the SC group, and that there is no significant difference or the difference is smaller than that of the SC group. Overall, the above results suggest that sepsis causes structural changes to the gut microbiota in rats.

According to the results of Fig. 4, species diversity was tested by Kruskal-Wallis test. The test showed the microorganisms with significant difference between SC group and CLP group, and between CLP group and CLP ADMSCs group. According to the above statistical test, the corresponding *P* value was obtained to judge whether there was significant difference among the three groups. We defined *P* < 0.05 as the species with significant difference. We analyzed the differences between the genus levels and different groups and found the important flora that affected the different groups. In septic rats, the proportion of Escherichia–Shigella (*P* = 0.01) related to lipopolysaccharide production increased, and the proportion of Akkermansia (*P* = 0.02) related to the regulation of intestinal mucosal thickness and the maintenance of intestinal barrier function decreased. Furthermore, the proportion of Firmicutes related to energy storage, such as Bacillus (*P* = 0.44), decreased. CLP-ADMSCs group compared with CLP group, the proportion of *Escherichia*–*Shigella* (*P* = 0.02) and the proportion of Pygmaiobacter (*P* = 0.03) decreased. But the proportion of *Akkermansia* (*P* = 0.02) increased.

## Discussion

This study shows that the gut microbiota of rats is significantly disturbed after sepsis and that the imbalance of the intestinal ecosystem plays an important role in the development of sepsis-induced ALI. Furthermore, treatment with ADMSCs can alleviate or even restore its normal microbial state and improve ALI. This finding suggests that the potential mechanism by which ADMSCs treat sepsis-induced ALI in rats involves the regulation of the gut microbiota.

MSCs have different tissue sources, but they have similar biological characteristics. ADMSCs and BMSCs are more valuable. ADMSCs have a similar regulatory effect with bone marrow-derived MSCs (BMSCs) in sepsis model, but ADMSCs are more easily obtained in clinical treatment [16]. ADMSCs are currently commercialized and easier to buy. More and importantly, we have been using ADMSCs, which has been published in our previous papers on sepsis [21, 74].

In order to study the role of the gut microbiota in rats with sepsis, our research team used 16S rDNA analysis technology for the first time to evaluate changes in the gut microbiota of rats after treatment with ADMSCs in the early stage of ALI caused by sepsis. The histological staining results showed that the lungs of rats with sepsis had serious lung injury, and the 16S rDNA sequencing results showed the characteristics of the gut microbiota disorder in the rats. Intervention with ADMSCs reduced organ damage, as well as inflammatory infiltration and hemorrhage, in the lungs, and the diversity of the flora and the content of pathogenic bacteria in rats with sepsis increased significantly. According to the recommendations in the official document of the American Thoracic Society (ATS), at least three of four main characteristics of ALI should be present in the ALI animal model, including histological evidence of tissue injury, alteration of the alveolar capillary barrier, inflammatory response, and physiological dysfunction [75]. In our results, pulmonary H&E showed typical inflammatory infiltration in sepsis-associated ALI, and decreased plasma IL-6/TNF-α through MSCs treatment, effectively alleviated inflammatory infiltration, and reduced bleeding and edema. In addition, we evaluated pulmonary edema by the lung wet/dry (W/D) weight ratios [21]. The percentage of apoptotic cells in CLP group was the highest by terminal deoxynucleotidyl transferase dUTP nick-end labeling (TUNEL) staining [21]. Here, we described the main relevant indicators of ALI that should be present. Some preclinical studies have shown that MSCs can significantly attenuate sepsis-associated ALI through immune regulation [76–79]. Some studies have shown that pulmonary H&E, inflammatory factors, and wet/dry (W/D) weight of lung tissue can evaluate the ALI model [80, 81]. In addition, we found that treatment with ADMSCs also reduced the expression of pro-inflammatory factors, such as TNF–α and IL-6, which is consistent with our previous studies [21, 74].

Firmicutes and Bacteroidetes are the main microflora in the human intestinal tract, and they are also the two most abundant microflora in the rat intestinal tract. Firmicutes and Bacteroidetes mainly participate in the regulation of fat and bile acid metabolism and the maintenance of the energy balance in the host [82]. Firmicutes help the body absorb and store energy from food, while Bacteroidetes do the opposite. This study showed that sepsis changed the gut microbiota and led to a significant decrease in food intake and energy storage in the rats. Therefore, the imbalance in the gut microbiota further aggravates the lung injury induced by sepsis. After ADMSC intervention in the SD rats, the proportion of Bacteroidetes and Firmicutes was partially corrected, energy intake and consumption was gradually balanced, the mortality of sepsis was effectively reduced, the inflammatory response in rats with sepsis was reduced, and the sepsis-induced ALI was improved.

Therefore, ADMSCs may reduce lung injury by blocking the release of inflammatory factors and may normalize the intestinal flora structure of septic rats, increasing beneficial bacteria, and reducing harmful bacteria. Overall, we studied the therapeutic effect of ADMSCs on ALI in sepsis and found that treatment with ADMSCs may relieve ALI and regulate the gut microbiota, so as to significantly improve the survival rate of rats with sepsis. Although the reduction of tissue damage was observed in our study, the specific relationship between the gut microbiota and lung tissue damage is still unclear. A study has shown that lower intestinal permeability has lower bacterial translocation and less epithelial damage [83]. The use of drugs can reduce inflammation and inhibit fibrosis by remodeling the microbiota to reduce damage [84]. In addition, some animal experiments and clinical studies have found that in mice and humans with sepsis-associated ALI, intestinal flora is transferred to the lung through the gut-lung axis [45, 53]. However, a study has found that when lung injury occurs, bacteria will enter the bloodstream, resulting in an increase in the load of intestinal flora [59]. Therefore, the potential mechanism of MSCs in the treatment of sepsis needs more exploration. There are other potential therapies for the clinics, such as microbiological guided therapy, including fecal microbiota transplantation (FMT) or probiotic therapy [85–87]. Previous studies of our team have found that the use of stem cells significantly reduces the mortality of septic rats and mice [21, 27, 74]. In future sepsis research, our team will continue to focus on the potential treatment and explore the treatment mechanism. A shortcoming of this study is that it is not yet verified whether ADMSCs may damage other organs in rats with sepsis at the same time, or whether they have a tendency to aggregate. This paper may provide potential drug treatment options for patients with sepsis, that is, to maintain the balance of the body’s own response by controlling the inflammatory response and maintaining the stability of the intestinal microecology.

## Conclusions

This is the first basic study on the changes of intestinal microorganisms caused by ADMSCs in the treatment of septic rats with ALI. The results show that ADMSCs relieve the ALI induced by CLP and improve the intestinal microflora, providing a potential mechanism for MSCs to treat sepsis.

## Supplementary information

**Additional file 1: Figure S1.** Macroscopic view of the rat model.

## Data Availability

The datasets used and/or analyzed in the present study are available from the corresponding author on reasonable request.

## Abbreviations

ADMSCs: Adipose-derived mesenchymal stem cells
ALI: Acute lung injury
H&E: Hematoxylin and eosin
ELISA: Enzyme-linked immunosorbent assay
PCoA: Principal coordinates analysis
MSCs: Mesenchymal stem cells
OTUs: Operational taxonomic units

## References

[1] Singer M, Deutschman CS, Seymour CW (2016). The Third International Consensus Definitions for Sepsis and Septic Shock (Sepsis-3)[J]. Jama.

[2] Annane D, Bellissant E, Cavaillon JM (2005). Septic shock. Lancet.

[3] Haak BW, Wiersinga WJ (2017). The role of the gut microbiota in sepsis. Lancet Gastroenterol Hepatol.

[4] Vincent JL, Marshall JC, Namendys-Silva SA (2014). Assessment of the worldwide burden of critical illness: the intensive care over nations (ICON) audit. Lancet Respir Med.

[5] Kwon WY, Suh GJ, Kim KS (2016). Niacin and selenium attenuate sepsis-induced lung injury by up-regulating nuclear factor erythroid 2-related factor 2 signaling. Crit Care Med.

[6] Potz BA, Sellke FW, Abid MR. Endothelial ROS and impaired myocardial oxygen consumption in sepsis-induced cardiac dysfunction. J Intensive Crit Care. 2016;2(1):20. 10.21767/2471-8505.100020 PMC4847432 27135058

[7] Schortgen F, Asfar P (2015). Update in sepsis and acute kidney injury 2014. Am J Respir Crit Care Med.

[8] Zhao L, An R, Yang Y (2015). Melatonin alleviates brain injury in mice subjected to cecal ligation and puncture via attenuating inflammation, apoptosis, and oxidative stress: the role of SIRT1 signaling. J Pineal Res.

[9] Abraham E, Singer M (2007). Mechanisms of sepsis-induced organ dysfunction. Crit Care Med.

[10] Fan YW, Jiang SW, Chen JM (2020). A pulmonary source of infection in patients with sepsis-associated acute kidney injury leads to a worse outcome and poor recovery of kidney function. World J Emerg Med.

[11] Zhu LL, Zhou Q (2018). Optimal infusion rate in antimicrobial therapy explosion of evidence in the last five years. Infect Drug Resist.

[12] Hebbar K, Rigby MR, Felner EI (2009). Neuroendocrine dysfunction in pediatric critical illness. Pediatr Crit Care Med.

[13] Irusen E, Matthews JG, Takahashi A (2002). p38 mitogen-activated protein kinase-induced glucocorticoid receptor phosphorylation reduces its activity: role in steroid-insensitive asthma. J Allergy Clin Immunol.

[14] Maumus M, Guerit D, Toupet K (2011). Mesenchymal stem cell-based therapies in regenerative medicine: applications in rheumatology. Stem Cell Res Ther.

[15] Zhu F, Guo GH, Chen W (2010). Effects of bone marrow-derived mesenchymal stem cells engraftment on vascular endothelial cell growth factor in lung tissue and plasma at early stage of smoke inhalation injury. World J Emerg Med.

[16] Naji A, Eitoku M, Favier B (2019). Biological functions of mesenchymal stem cells and clinical implications. Cell Mol Life Sci.

[17] Johnson CL, Soeder Y, Dahlke MH (2017). Concise review: Mesenchymal stromal cell-based approaches for the treatment of acute respiratory distress and sepsis syndromes. Stem Cells Transl Med.

[18] Krasnodembskaya A, Samarani G, Song Y (2012). Human mesenchymal stem cells reduce mortality and bacteremia in gram-negative sepsis in mice in part by enhancing the phagocytic activity of blood monocytes. Am J Physiol Lung Cell Mol Physiol.

[19] de Witte SFH, Luk F, Sierra Parraga JM (2018). Immunomodulation by therapeutic mesenchymal stromal cells (MSC) is triggered through phagocytosis of MSC by monocytic cells. Stem Cells.

[20] Mei SH, Haitsma JJ, Dos Santos CC (2010). Mesenchymal stem cells reduce inflammation while enhancing bacterial clearance and improving survival in sepsis. Am J Respir Crit Care Med.

[21] Ding XF, Liang HY, Sun JY (2019). Adipose-derived mesenchymal stem cells ameliorate the inflammatory reaction in CLP-induced septic acute lung injury rats via sTNFR1.

[22] Sun CK, Yen CH, Lin YC (2011). Autologous transplantation of adipose-derived mesenchymal stem cells markedly reduced acute ischemia-reperfusion lung injury in a rodent model. J Transl Med.

[23] Le Blanc K, Tammik L, Sundberg B (2003). Mesenchymal stem cells inhibit and stimulate mixed lymphocyte cultures and mitogenic responses independently of the major histocompatibility complex. Scand J Immunol.

[24] Gonzalez-Rey E, Anderson P, Gonzalez MA (2009). Human adult stem cells derived from adipose tissue protect against experimental colitis and sepsis. Gut.

[25] Gonzalez MA, Gonzalez-Rey E, Rico L (2009). Adipose-derived mesenchymal stem cells alleviate experimental colitis by inhibiting inflammatory and autoimmune responses. Gastroenterology.

[26] Nemeth K, Leelahavanichkul A, Yuen PS (2009). Bone marrow stromal cells attenuate sepsis via prostaglandin E(2)-dependent reprogramming of host macrophages to increase their interleukin-10 production. Nat Med.

[27] Sun XY, Ding XF, Liang HY (2020). Efficacy of mesenchymal stem cell therapy for sepsis: a meta-analysis of preclinical studies. Stem Cell Res Ther.

[28] Pedrazza L, Cubillos-Rojas M, de Mesquita FC (2017). Mesenchymal stem cells decrease lung inflammation during sepsis, acting through inhibition of the MAPK pathway. Stem Cell Res Ther.

[29] Shi Y, Wang Y, Li Q (2018). Immunoregulatory mechanisms of mesenchymal stem and stromal cells in inflammatory diseases. Nat Rev Nephrol.

[30] Ikarashi S, Tsuchiya A, Kawata Y (2019). Effects of human adipose tissue-derived and umbilical cord tissue-derived mesenchymal stem cells in a dextran sulfate sodium-induced mouse model. Biores Open Access.

[31] Li D, Wang P, Wang P (2016). The gut microbiota: a treasure for human health. Biotechnol Adv.

[32] Hooper LV, Macpherson AJ (2010). Immune adaptations that maintain homeostasis with the intestinal microbiota. Nat Rev Immunol.

[33] Dorman G, Kocsis-Szommer K, Spadoni C (2007). MMP inhibitors in cardiac diseases: an update. Recent Pat Cardiovasc Drug Discov.

[34] Luo JX, Zhang Y, Hu XY, Yu C. The effect of modified sini decoction on survival rates of patients with hepatitis B virus related acute-on-chronic liver failure. Evid Based Complement Alternat Med. 2019;2019:2501847. 10.1155/2019/2501847 PMC6409021 30915144

[35] Stewart CJ, Embleton ND, Marrs ECL (2017). Longitudinal development of the gut microbiome and metabolome in preterm neonates with late onset sepsis and healthy controls. Microbiome.

[36] Haak BW, Prescott HC, Wiersinga WJ (2018). Therapeutic potential of the gut microbiota in the prevention and treatment of sepsis. Front Immunol.

[37] West CE, Renz H, Jenmalm MC (2015). The gut microbiota and inflammatory noncommunicable diseases: associations and potentials for gut microbiota therapies. J Allergy Clin Immunol.

[38] Klingensmith NJ, Coopersmith CM (2016). The gut as the motor of multiple organ dysfunction in critical illness. Crit Care Clin.

[39] Yoshihisa A (2018). Altered gut flora and gut microbiome-derived metabolites in heart failure patients in the compensated and decompensated phases. Circ J.

[40] Li B, Yin GF, Wang YL, Tan YM, Huang CL, Fan XM. Impact of fecal microbiota transplantation on TGF-β1/Smads/ERK signaling pathway of endotoxic acute lung injury in rats. 3 Biotech. 2020;10(2):52. 10.1007/s13205-020-2062-4 PMC6971213 32015948

[41] Tang SY, Zhang SW, Zhang J, et al. Effect of early fluid resuscitation combined with low dose cyclophosphamide on intestinal barrier function in severe sepsis rats. Drug Deliv Transl Res. 2018;8(5):1254–64. 10.1007/s13346-018-0573-x 30112606

[42] Rittirsch D, Huber-Lang MS, Flierl MA (2009). Immunodesign of experimental sepsis by cecal ligation and puncture. Nat Protoc.

[43] Costa EL, Schettino IA, Schettino GP (2006). The lung in sepsis: guilty or innocent?. Endocr Metab Immune Disord Drug Targets.

[44] Dickson RP, Schultz MJ, van der Poll T (2020). Lung microbiota predict clinical outcomes in critically ill patients. Am J Respir Crit Care Med.

[45] Mukherjee S, Hanidziar D (2018). More of the gut in the lung: how two microbiomes meet in ARDS. Yale J Biol Med.

[46] Poroyko V, Meng F, Meliton A (2015). Alterations of lung microbiota in a mouse model of LPS-induced lung injury. Am J Physiol Lung Cell Mol Physiol.

[47] Yin Y, Hountras P, Wunderink RG (2017). The microbiome in mechanically ventilated patients. Curr Opin Infect Dis.

[48] Molyneaux PL, Cox MJ, Wells AU (2017). Changes in the respiratory microbiome during acute exacerbations of idiopathic pulmonary fibrosis. Respir Res.

[49] Barcik W, Boutin RCT, Sokolowska M (2020). The role of lung and gut microbiota in the pathology of asthma. Immunity.

[50] Beck JM, Young VB, Huffnagle GB (2012). The microbiome of the lung. Transl Res.

[51] Mammen MJ, Sethi S (2016). COPD and the microbiome. Respirology.

[52] Schmitt FCF, Lipinski A, Hofer S, et al. Pulmonary microbiome patterns correlate with the course of the disease in patients with sepsis-induced ARDS following major abdominal surgery. J Hosp Infect. 2020;105(3):438–46. 10.1016/j.jhin.2020.04.028 32339614

[53] Dickson RP, Singer BH, Newstead MW (2016). Enrichment of the lung microbiome with gut bacteria in sepsis and the acute respiratory distress syndrome. Nat Microbiol.

[54] Bassis CM, Erb-Downward JR, Dickson RP (2015). Analysis of the upper respiratory tract microbiotas as the source of the lung and gastric microbiotas in healthy individuals. mBio.

[55] Dickson RP, Erb-Downward JR, Huffnagle GB (2014). Towards an ecology of the lung: new conceptual models of pulmonary microbiology and pneumonia pathogenesis. Lancet Respir Med.

[56] Fonkou MD, Dufour JC, Dubourg G (2018). Repertoire of bacterial species cultured from the human oral cavity and respiratory tract. Future Microbiol.

[57] Sherwin R, Winters ME, Vilke GM (2017). Does early and appropriate antibiotic administration improve mortality in emergency department patients with severe Sepsis or septic shock?. J Emerg Med.

[58] Di Cicco M, Pistello M, Jacinto T (2018). Does lung microbiome play a causal or casual role in asthma?. Pediatr Pulmonol.

[59] Sze MA, Tsuruta M, Yang SW (2014). Changes in the bacterial microbiota in gut, blood, and lungs following acute LPS instillation into mice lungs. PLoS One.

[60] Dickson RP, Erb-Downward JR, Freeman CM (2015). Spatial variation in the healthy human lung microbiome and the adapted island model of lung biogeography. Ann Am Thorac Soc.

[61] Sung JY, Hwang Y, Shin MH (2018). Utility of conventional culture and MALDI-TOF MS for identification of microbial communities in Bronchoalveolar lavage fluid in comparison with the GS junior next generation sequencing system. Ann Lab Med.

[62] Ojima M, Motooka D, Shimizu K (2016). Metagenomic analysis reveals dynamic changes of whole gut microbiota in the acute phase of intensive care unit patients. Dig Dis Sci.

[63] Zhao T, Pan B, Alam HB (2016). Protective effect of Cl-amidine against CLP-induced lethal septic shock in mice. Sci Rep.

[64] Ding XF, Sun M, Guan FX (2017). Prenatal exposure to LPS alters the intrarenal RAS in offspring, which is ameliorated by adipose tissue-derived mesenchymal stem cells. Am J Hypertens.

[65] Logue JB, Stedmon CA, Kellerman AM, et al. Experimental insights into the importance of aquatic bacterial community composition to the degradation of dissolved organic matter. ISME J. 2016;10(3):533–45. 10.1038/ismej.2015.131 PMC4817675 26296065

[66] Walters W, Hyde ER, Berg-Lyons D, et al. Improved bacterial 16S rRNA gene (V4 and V4-5) and fungal internal transcribed spacer marker gene primers for microbial community surveys. mSystems. 2015;1(1):e00009–15. 10.1128/mSystems.00009-15 PMC5069754 27822518

[67] Takai K, Horikoshi K (2000). Rapid detection and quantification of members of the archaeal community by quantitative PCR using fluorogenic probes. Appl Environ Microbiol.

[68] Xie J, Liu Y, Chen B, et al. Ganoderma lucidumpolysaccharide improves rat DSS-induced colitis by altering cecal microbiota and gene expression of colonic epithelial cells. Food Nutr Res. 2019;63. 10.29219/fnr.v63.1559. 10.29219/fnr.v63.1559 PMC6387425 30814921

[69] Vega-Magana N, Delgado-Rizo V, Garcia-Benavides L (2018). Bacterial translocation is linked to increased intestinal IFN-gamma, IL-4, IL-17, and mucin-2 in cholestatic rats. Ann Hepatol.

[70] Shogan BD, Smith DP, Christley S (2014). Intestinal anastomotic injury alters spatially defined microbiome composition and function. Microbiome.

[71] Alam A, Leoni G, Quiros M, et al. The microenvironment of injured murine gut elicits a local pro-restitutive microbiota. Nat Microbiol. 2016;1:15021. 10.1038/nmicrobiol.2015.21 PMC5076466 27571978

[72] Berry D, Stecher B, Schintlmeister A (2013). Host-compound foraging by intestinal microbiota revealed by single-cell stable isotope probing. Proc Natl Acad Sci U S A.

[73] Zhang Z, Wu X, Cao S, et al. Chlorogenic acid ameliorates experimental colitis by promoting growth of Akkermansia in mice. Nutrients. 2017;9(7):677. 10.3390/nu9070677 PMC5537792 28661454

[74] Liang H, Ding X, Yu Y (2019). Adipose-derived mesenchymal stem cells ameliorate acute liver injury in rat model of CLP induced-sepsis via sTNFR1. Exp Cell Res.

[75] Lian J, Lin J, Zakaria N, Yahaya BH. Acute lung injury: disease modelling and the therapeutic potential of stem cells [published online ahead of print, 2020 May 19]. Adv Exp Med Biol. 2020. 10.1007/5584_2020_538. 10.1007/5584_2020_538 32424492

[76] Hao Q, Zhu YG, Monsel A (2015). Study of bone marrow and embryonic stem cell-derived human mesenchymal stem cells for treatment of Escherichia coli endotoxin-induced acute lung injury in mice. Stem Cells Transl Med.

[77] Li J, Li D, Liu X (2012). Human umbilical cord mesenchymal stem cells reduce systemic inflammation and attenuate LPS-induced acute lung injury in rats. J Inflamm (Lond).

[78] Matthay MA, Thompson BT, Read EJ (2010). Therapeutic potential of mesenchymal stem cells for severe acute lung injury. Chest.

[79] Ho MS, Mei SH, Stewart DJ (2015). The immunomodulatory and therapeutic effects of mesenchymal stromal cells for acute lung injury and sepsis. J Cell Physiol.

[80] Li JW, Wu X (2015). Mesenchymal stem cells ameliorate LPS-induced acute lung injury through KGF promoting alveolar fluid clearance of alveolar type II cells. Eur Rev Med Pharmacol Sci.

[81] Liu L, He H, Liu A (2015). Therapeutic effects of bone marrow-derived mesenchymal stem cells in models of pulmonary and extrapulmonary acute lung injury. Cell Transplant.

[82] Pan H, Guo R, Zhu J, et al. A gene catalogue of the Sprague-Dawley rat gut metagenome. Gigascience. 2018;7(5):giy055. 10.1093/gigascience/giy055 PMC5967468 29762673

[83] Liu T, Wu Y, Wang L, et al. A more robust gut microbiota in calorie-restricted mice is associated with attenuated intestinal injury caused by the chemotherapy drug cyclophosphamide. mBio. 2019;10(2):e02903–18. 10.1128/mBio.02903-18 PMC6414708 30862756

[84] Zhao Z, Cheng W, Qu W (2020). Antibiotic alleviates radiation-induced intestinal injury by remodeling microbiota, reducing inflammation, and inhibiting fibrosis. ACS Omega.

[85] Schuijt TJ, Lankelma JM, Scicluna BP, et al. The gut microbiota plays a protective role in the host defence against pneumococcal pneumonia. Gut. 2016;65(4):575–83. 10.1136/gutjnl-2015-309728 PMC4819612 26511795

[86] Dickson RP (2018). The lung microbiome and ARDS. It is time to broaden the model. Am J Respir Crit Care Med.

[87] Nakov R, Segal JP, Settanni CR, et al. Microbiome: what intensivists should know. Minerva Anestesiol. 2020;86(7):777–85. 10.23736/S0375-9393.20.14278-0 32368882

