# Supplementary figures and images for: Adipose-derived mesenchymal stem cells attenuate acute lung injury and improve the gut microbiota in septic rats
Source: Stem Cell Res Ther. 2020 Sep 7;11:384. doi: 10.1186/s13287-020-01902-5 (PMC7487801; doi:10.1186/s13287-020-01902-5)

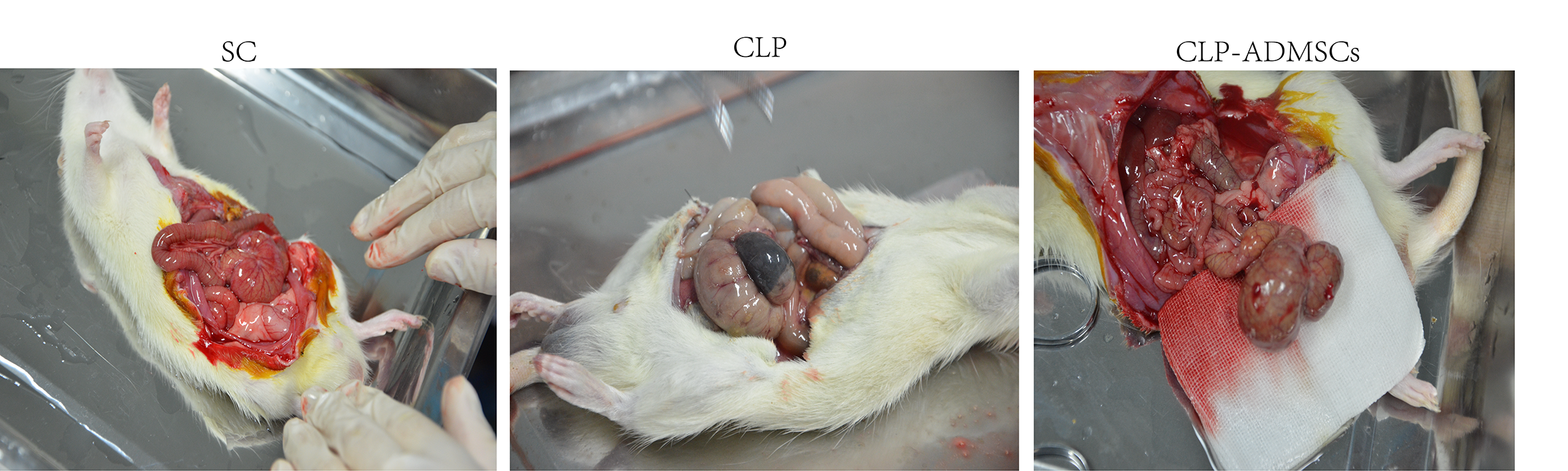

Supplement: Supplementary file 1 — Additional file 1: Figure S1. Macroscopic view of the rat model. [file 13287_2020_1902_MOESM1_ESM.tif]
